# On global minimizers of repulsive–attractive power-law interaction energies

**DOI:** 10.1098/rsta.2013.0399

**Published:** 2014-11-13

**Authors:** José Antonio Carrillo, Michel Chipot, Yanghong Huang

**Affiliations:** 1 Department of Mathematics, Imperial College London, South Kensington, London SW7 2AZ, UK; 2 Institut für Mathematik, Angewandte Mathematik, Winterthurerstrasse 190, 8057 Zürich, Switzerland

**Keywords:** interaction energy, global discrete minimizers, swarming models

## Abstract

We consider the minimization of the repulsive–attractive power-law interaction energies that occur in many biological and physical situations. We show the existence of global minimizers in the discrete setting and obtain bounds for their supports independently of the number of Dirac deltas in a certain range of exponents. These global discrete minimizers correspond to the stable spatial profiles of flock patterns in swarming models. Global minimizers of the continuum problem are obtained by compactness. We also illustrate our results through numerical simulations.

## 1. Introduction

Let *μ* be a probability measure on 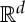. We are interested in minimizing the interaction potential energy defined by
1.1
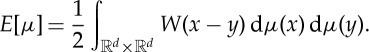

**Figure 1.**
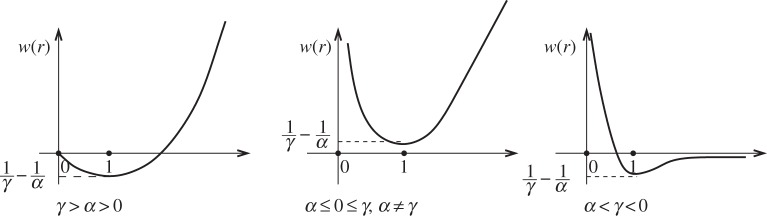
Three different behaviours of *w*(*r*)=*r*^*γ*^/*γ*−*r*^*α*^/*α*, *γ*>*α*.

Here, *W* is a repulsive–attractive power-law potential,
1.2
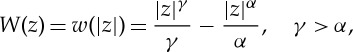

with the understanding that 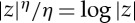 for *η*=0. Moreover, we define 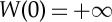 if *α*≤0. This is the simplest possible potential that is repulsive in the short range and attractive in the long range. Depending on the signs of the exponents *γ* and *α*, the behaviour of the potential is depicted in figure 1. Because this potential *W* is bounded from below by *w*(1)=1/*γ*−1/*α*, the energy *E*[*μ*] always makes sense, with possibly positive infinite values.

The minimizers of the energy *E*[*μ*] are related to stationary states for the aggregation equation *ρ*_*t*_=∇⋅(*ρ*∇W*ρ) studied in [1–5] with repulsive–attractive potentials [6–12]. The set of local minimizers of the interaction energy, in both the discrete setting of empirical measures (equal-mass Dirac deltas) and the continuum setting of general probability measures, can exhibit rich complicated structure as studied numerically in [13,12]. In fact, it is shown in [12] that the dimensionality of the support of local minimizers of (1.1) depends on the strength of the repulsion at zero of the potential *W*. For instance, as the repulsion at the origin becomes stronger (i.e. *α* gets smaller) in three dimensions, the support of the local minimizer is concentrated on points, curves, surfaces and eventually some sets of non-zero Lebesgue measure.

From the viewpoint of applications, these models with non-local interactions are ubiquitous in the literature. Convex attractive potentials appear in granular media [1,2,14,15]. More sophisticated potentials such as (1.2) are included to take into account short-range repulsion and long-range attraction in kinematic models of the collective behaviour of animals; see [13,16–19] and the references therein. The minimization of the interaction energy in the discrete setting is of paramount importance for the structure of virus capsids [20], for self-assembly materials in chemical engineering design [21–23] and for flock patterns in animal swarms [24–26].

Despite the efforts in understanding the qualitative behaviour of stationary solutions to the aggregation equation *ρ*_*t*_=∇⋅(*ρ*∇W*ρ) and the structure of local minimizers of the interaction energy *E*[*μ*], there are no general results addressing the global minimization of *E*[*μ*] in the natural framework of probability measures. See [27] for a recent analysis of this question in the more restricted set of bounded or binary densities. Here, we first try to find solutions in the restricted set of atomic measures.

The interest in understanding the global discrete minimizers of the interaction energy is not purely mathematical. The discrete global minimizers give the spatial profile of typical flocking patterns obtained in simplified models for social interaction between individuals as in [28,13] based on the famous three-zone models [29,30]. Moreover, owing to the recent nonlinear stability results in [26], we know now that the stability properties of the discrete global minimizer as the stationary solution of the first-order ordinary differential equation model

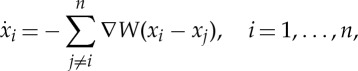

lead to the stability properties of the flock profiles for the second-order model in swarming introduced in [17] or additional alignment mechanisms such as the Cucker–Smale interaction [31,32]; see also [28] and the discussion therein.

Our objective is to show the existence of global minimizers of the interaction energy defined on probability measures under some conditions on the exponents. Our approach starts with the discrete setting by showing the qualitative properties of the global minimizers in the set of equal-mass Dirac deltas. These discrete approximations are used extensively in materials science and variational calculus with hard-core potentials [33–36] in order to understand the crystallization phenomenon. However, these discrete approximations with soft potentials such as (1.2) are more difficult; apart from various properties of the minimizers [8,13,17,24], the existence as well as the convergence of these discrete minimizers are not established in general. In a certain range of exponents, we prove that the diameter of the support of the discrete minimizers does not depend on the number of Dirac deltas. This result, together with standard compactness arguments, results in our desired global minimizers among probability measures.

In fact, our strategy to show the confinement of discrete minimizers is in the same spirit as the proof of the confinement of solutions of the aggregation equation in [37,38]. In our case, the ideas behind the proof in §2 are based on convexity-type arguments in the range of exponents *γ*>*α*≥1 to show the uniform bound in the diameter of global minimizers in the discrete setting. Section 3 is devoted to more refined results in one dimension. We show that, for very repulsive potentials, the bounds on the diameter are not uniform in the number of Dirac deltas, complemented by numerical simulations. In the range of exponents *γ*>1>*α*, the minimizers turn out to be unique (up to translation), analogous to the simplified displacement convexity in one dimension. In the special case *γ*=2 and *α*=1, we can find the minimizers and show the convergence to the continuous minimizer explicitly.

## 2. Existence of global minimizers

We first consider the discrete setting, where *μ* is a convex combination of Dirac deltas, i.e.

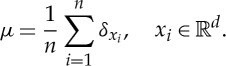

Setting
2.1
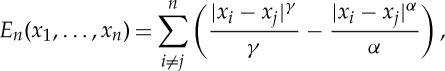

for such a *μ*, one has *E*[*μ*]=(1/(2*n*^2^))*E*_*n*_(*x*_1_,…,*x*_*n*_). In the definition of the energy, we can include the self-interaction for non-singular cases, *α*>0, because the two definitions coincide. Fixing 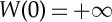 for singular kernels makes *W* upper semi-continuous, and the self-interaction must be excluded to have finite energy configurations.

Let us remark that, owing to the translational invariance of the interaction energy, minimizers of the interaction energy *E*[*μ*] can be expected only up to translations. Moreover, when the potential is radially symmetric, as in our case, then any isometry in 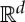 also leaves invariant the interaction energy. These invariances are also inherited by the discrete counterpart *E*_*n*_(*x*_1_,…,*x*_*n*_). We first consider the minimizers of *E*_*n*_(*x*) among all 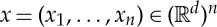, and then the convergence to the global minimizers of *E*[*μ*] as *n* goes to infinity.

### (a) Existence of minimizer: discrete setting

Let us consider, for *α*<*γ*, the derivative of the radial potential,

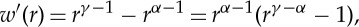

which obviously vanishes for *r*=1 and for *r*=0 when *α*>1. We conclude, from the sign of the derivatives, that *w*(*r*) always attains a global minimum at *r*=1. There are, following the values of *α*<*γ*, three types of behaviour for *w*, which are shown in figure 1. In all three cases, *E*_*n*_ is bounded from below because

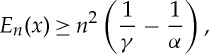

with the understanding that 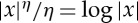 for *η*=0. We set
2.2
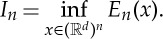

Using the translational invariance of *E*_*n*_(*x*_1_,…,*x*_*n*_), we can assume without loss of generality that *x*_1_=0, which we do throughout this subsection. First, we have lemma 2.1 showing that *I*_*n*_ is achieved, which can be proved by discussing different ranges of the exponents *γ* and *α*.

**Lemma 2.1**

*For any finite n (≥2), the minimum value I_n_ is obtained for some discrete minimizers in* 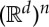.

**Proof.**

*The case* 0<*α*<*γ*. We claim that
2.3
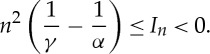

Indeed, consider 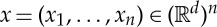 such that *x*_1_,…,*x*_*n*_ are aligned and ∣*x*_*i*_−*x*_*i*+1_∣=1/*n*. Then, for any *i*,*j*, one has 0<∣*x*_*i*_−*x*_*j*_∣≤1 and *w*(∣*x*_*i*_−*x*_*j*_∣)<0. Therefore, (2.3) follows.

Let us show that the infimum *I*_*n*_ is achieved. Let 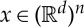. Set 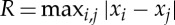. A minimizer is sought among the points such that *E*_*n*_(*x*)<0, and one has for such a point

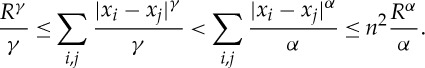

This implies the upper bound
2.4
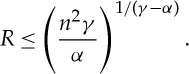

Thus, because *x*_1_=0, all the *x*_*i*_ have to be in the ball of centre 0 and radius (*n*^2^*γ*/*α*)^1/(*γ*−*α*)^, i.e. *x* has to be in a compact set of 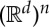. Because *E*_*n*_(*x*) is continuous, the infimum *I*_*n*_ is achieved. Note that the bound on the radius, where all Dirac deltas are contained, depends *a priori* on *n*.

*The case* *α*≤0≤*γ* *and* *α*≠*γ*. In this case, 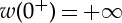 and 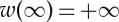. We minimize among all *x* such that *x*_*i*_≠*x*_*j*_ for *i*≠*j*. Note that *w* and *I*_*n*_ are both positive. Because 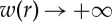 as *r*→0 or 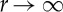, there exist *a*_*n*_,*b*_*n*_>0 such that

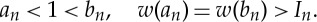

Let 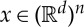. If, for a pair *i*,*j*, one has
2.5
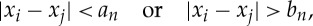

then one has *E*_*n*_(*x*)>*I*_*n*_. Thus, the infimum (2.2) is not achieved among the points *x* satisfying (2.5) but among those in

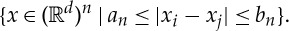

Because the set above is compact, being closed and contained in (*B*(0,*b*_*n*_))^*n*^ because *x*_1_=0, the infimum *I*_*n*_ is achieved.

*The case* *α*<*γ*<0. In this case, *I*_*n*_<0. Indeed, it is enough to choose

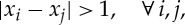

to obtain *E*_*n*_(*x*)<0. Because 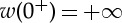, we minimize *E*_*n*_ among the points *x* such that *x*_*i*_≠*x*_*j*_, *i*≠*j*. Thus, the summation is over *n*^2^−*n* pairs (*i*,*j*). Denote by 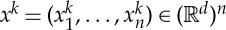 a minimizing sequence of *E*_*n*_. Because 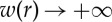 as *r*→0, there exists a number *a*_*n*_<1 such that

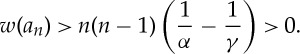

If, for a pair (*i*,*j*), one has 
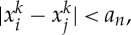
 then

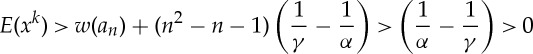

and *x*^*k*^ cannot be a minimizing sequence. So, without loss of generality, we may assume that 
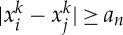
, ∀ *i*,*j*. Let us denote by *y*_1_,…,*y*_*d*_ the coordinates in 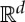. Without loss of generality, we can assume by relabelling and isometry invariance that for every *k* one has

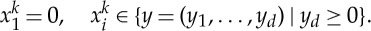

Suppose that 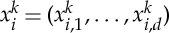 and the numbering of the points is done in such a way that 
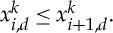

We next claim that one can assume that 
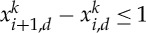
, ∀ *i*. Indeed, if not, let *i*_0_ be the first index such that

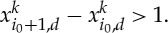

Let us leave the first 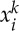 until *i*_0_ unchanged and for *i*>*i*_0_ replace 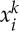 by

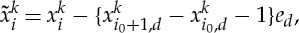

where *e*_*d*_ is the *d*-vector of the canonical basis of 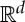, i.e. we shift 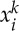 down in the direction *e*_*d*_ by 
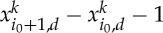
. Denote by 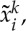 the new sequence obtained in this manner. One has

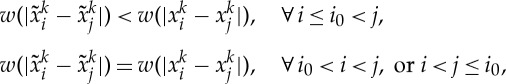

and thus one has obtained a minimizing sequence with

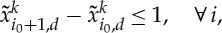

i.e. 
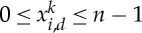
, for all *i*.

Repeating this process in the other directions, one can assume without loss of generality that
2.6
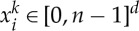

for all *k*, i.e. that *x*^*k*^ is in a compact subset of 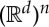, and extracting a convergent subsequence, we obtain our desired minimizer in [0,*n*−1]^*d*^. ▪

### (b) Existence of minimizer: general measures

The estimates (2.4) and (2.6) give estimates for the support of a minimizer of (2.2). However, these estimates depend on *n*. We show now that the diameter of any minimizer of (2.2) can sometimes be bounded independently of *n*.

**Theorem 2.2**

*Suppose that 1≤α<γ. Then, the diameter of any global minimizer of E*_*n*_ *achieving the infimum in (2.2) is bounded independently of n.*

**Proof.**

At a point 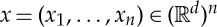 where the minimum of *E*_*n*_ is achieved, one has

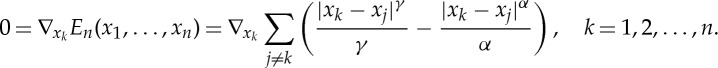

Because ∇_*x*_(∣*x*∣^*η*^/*η*)=∣*x*∣^*η*−2^*x*, we obtain
2.7
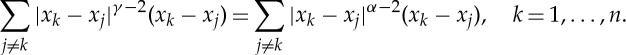

Suppose the points are labelled in such a way that

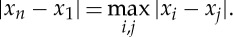

Then for *k*=1 and *n* in (2.7), we obtain

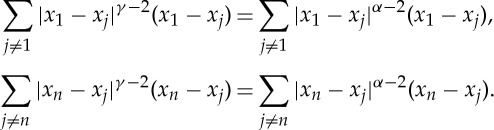

By subtraction, this leads to

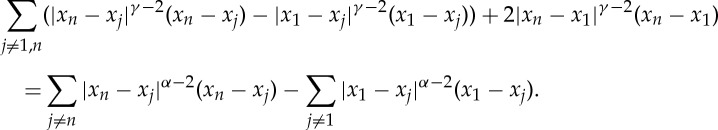

Taking the scalar product of both sides with *x*_*n*_−*x*_1_, we obtain

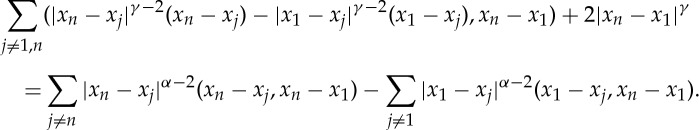

For *γ*≥2, there exists a constant *C*_*γ*_>0 such that (see [39])
2.8
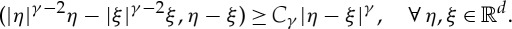

Note that this is nothing other than the modulus of convexity (in the sense of [2]) of the potential ∣*x*∣^*γ*^. Thus, estimating from above, we derive

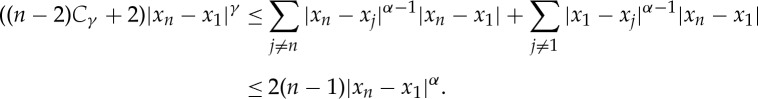

Thus, if *a*∧*b* denotes the minimum of two numbers *a* and *b*, we derive

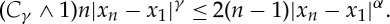

That is

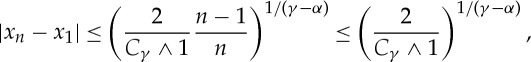

which proves the theorem in the case *γ*≥2. In the case where 1<*γ*<2, one can replace (2.8) with

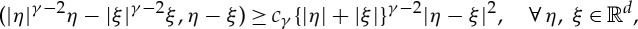

for some constant *c*_*γ*_ (see [39]). We obtain, arguing as above,

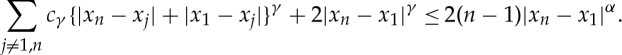

Now because *γ*−2<0, ∣*x*_*n*_−*x*_*j*_∣<∣*x*_*n*_−*x*_1_∣ and ∣*x*_1_−*x*_*j*_∣<∣*x*_*n*_−*x*_1_∣, we derive that

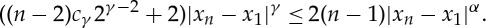

We thus obtain the bound

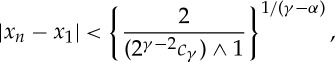

which completes the proof of the theorem. ▪

As a direct consequence of this bound being independent of the number of Dirac deltas, we can prove the existence of global minimizers in the continuous setting.

**Theorem 2.3**

*Suppose that 1≤α<γ. Then, global minimizers associated with the global minimum of E*_*n*_*(x) with zero centre of mass converge as* 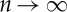 *towards a global minimizer among all probability measures with bounded moments of order γ of the interaction energy E[μ] in (1.1).*

**Proof.**

Let 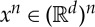 be a minimizer of (2.1) and

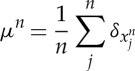

be the associated discrete measure. From theorem 2.2, the radius of the supports of the measures *μ*^*n*^ is bounded uniformly in *n* by *R*, provided that the centre of the mass 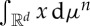 is normalized to be the origin. By Prokhorov's theorem [40], {*μ*^*n*^} is compact in the weak-* topology of measures and also in the metric space induced by *γ*-Wasserstein distance *d*_*γ*_ between probability measures (see [41,42] for definition and basic properties). Then, there is a measure μ* supported on *B*(0,*R*) such that

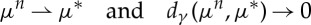

as *n* goes to infinity. Note that the notion of convergence of a sequence of probability measures in *d*_*γ*_ is equivalent to weak convergence of the measures plus convergence of the moments of order *γ*; see [42], ch. 9. Let *ν* be any probability measure on 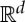 with bounded moment of order *γ*; then 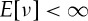. Moreover, there is a sequence of discrete measures *ν*^*n*^ of the form

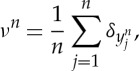

such that *d*_*γ*_(*ν*^*n*^,*ν*)→0, and thus 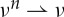; see [41,42]. By the definition of *E*_*n*_ in (2.2), we deduce

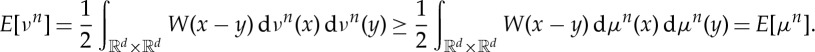

On the other hand, because

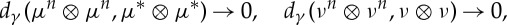

as 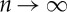, and the function *w*(*x*−*y*)=∣*x*−*y*∣^*γ*^/*γ*−∣*x*−*y*∣^*α*^/*α* is Lipschitz continuous on bounded sets in 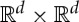 with growth of order *γ* at infinity, then

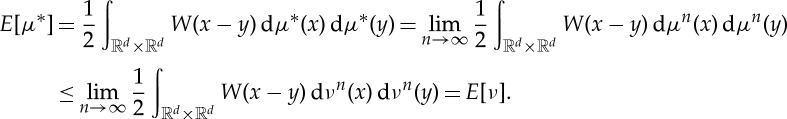

Therefore, μ* must be a global minimizer of *E*[*μ*] in the set of probability measures with bounded moments of order *γ*. ▪

**Remark 2.4**

The convergence of the minimizers of *E*_*n*_ can be proved also in the general framework of *Γ*-convergence, a well-known technique of variational convergence of sequences of functionals. This approach was implemented successfully to show the rescaled configurations to the Wulff shape [35] and general measure quantization of power repulsion–attraction potentials [43].

**Remark 2.5**

Global minimizers of the energy in the continuum setting might be a convex combination of a finite number of Dirac deltas. Numerical experiments suggest that this is always the case in the range 2<*α*<*γ*. It is an open problem in this range to show that global minimizers in the discrete case do not change (except symmetries) for *n* large enough and coincide with global minimizers of the continuum setting.

**Remark 2.6**

The range of exponents 1≤*α*<*γ* in theorem 2.3 can be extended to *γ*≥1 and *γ*>*α*>0, using uniform bounds on the *γ*th moments of the minimizers. First, if 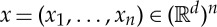 is a minimizer of *E*_*n*_ with centre of mass at the origin, then by (2.3) and Hölder inequality

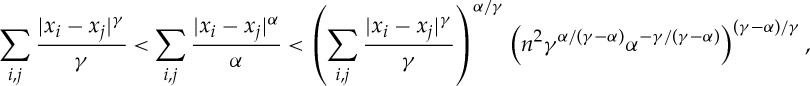

or equivalently

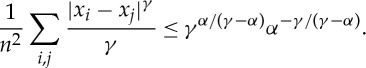

Because the function *Φ*(*x*)=∣*x*−*y*∣^*γ*^ is convex for any *γ*≥1 and 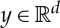, Jensen's inequality implies that

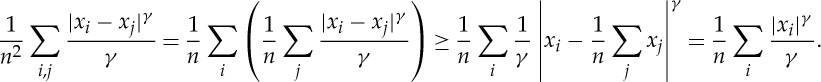

As a consequence, we obtain a uniform bound on the *γ*th moment of the discrete minimizers. As a result, the minimizing sequence corresponding to the associated atomic measures is tight, leading also towards a global minimizer of *E*[*μ*].

## 3. Further remarks in one dimension

Here, we concentrate on the one-dimensional case (*d*=1) for more refined properties.

### (a) Confinement of discrete global minimizers

We check first how sharp are the conditions on the exponents of the potential to obtain the confinement of global discrete minimizers independently of *n*. In fact, when the potential is very repulsive at the origin, we can show that a uniform bound in *n* of the diameter of the global minimizers in the discrete setting does not hold. If *x* is a minimizer of *E*_*n*_(*x*), we always assume that the labelling of the *x*_*i*_ is in increasing order: *x*_1_≤*x*_2_≤⋯≤*x*_*n*_.

**Theorem 3.1**

*Suppose α<γ<0 and α<−2. If x is a minimizer of E*_*n*_*, then there exists a constant C*_*α*,*γ*_ *such that, for n large enough,*

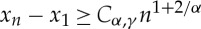

*holds.*

**Proof.**

Set *C*=1/*α*−1/*γ*>0. Denote by *a*_*n*_ the unique element of 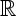 such that
3.1
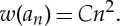

If *x* is a minimizer of *E*_*n*_, we claim that
3.2
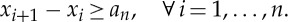

Indeed, otherwise,

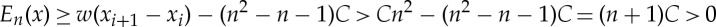

and we know that in this case *E*_*n*_<0. From (3.1), we derive

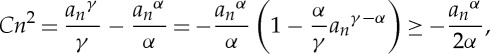

for *n* large enough (recall that *a*_*n*_→0 when 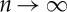). It follows that

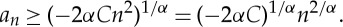

Combining this with (3.2), we obtain

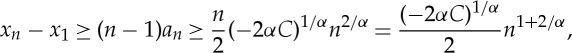

for *n* large enough, proving the desired estimate with *C*_*α*,*γ*_=(−2*αC*)^1/*α*^/2. ▪

**Figure 2.**
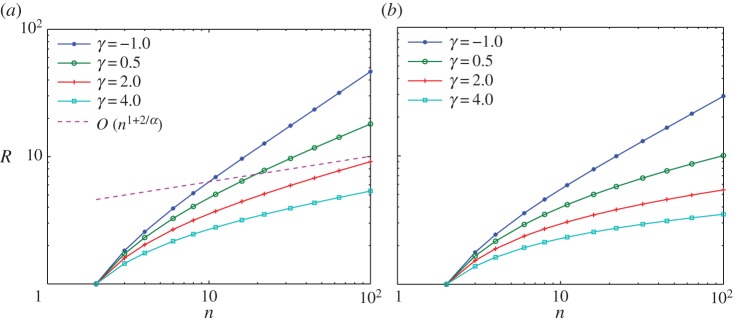
The dependence of the diameter 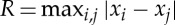 on the number of particles *n*: (*a*) *α*=−2.5; (*b*) *α*=−1.5. (Online version in colour.)

This property for the minimizers of this very repulsive case is similar to H-stability in statistical mechanics [44], where the minimal distance between two particles is expected to be constant when *n* is large, and crystallization occurs. This also suggests that the lower bound *O*(*n*^1+2/*α*^) is not sharp, which is verified in figure 2.

In fact, numerical experiments in [12,38] suggest that confinement happens for −1<*α*<1. It is an open problem to obtain a uniform bound in the support of the discrete minimizers as in §2 in this range. In the range *α*≤−1, our numerical simulations suggest that spreading of the support happens for all *γ*, with a decreasing spreading rate as *γ* increases. For hard-core potentials considered in [33–36], the crystallization can be rescaled to a macroscopic cluster with uniform density; however, the scaling relation seems to have a more delicate dependence on the parameters when *α*≤2.

### (b) Uniqueness of global minimizers

We turn now to the issue of uniqueness (up to isometry) of global discrete and continuum minimizers. In general, a large number of discrete minimizers (partially due to symmetries) are expected, and the uniqueness can be shown only in the macroscopic limit [35]. If *x* is a minimizer of *E*_*n*_(*x*), we can always assume at the expense of a translation that the centre of mass is zero, that is (*x*_1_+⋯+*x*_*n*_)/*n*=0. Let us recall that

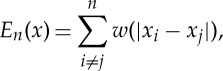

with the convention that *x*_*i*_≠*x*_*j*_ when *i*≠*j*, *α*<0.

**Lemma 3.2**

*Suppose that α≤1, γ≥1 and α<γ. Let x, y be two points of* 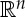 *such that*
3.3a
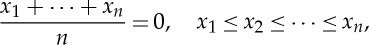

*and*
3.3b
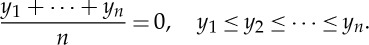

*Then*

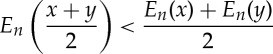

*unless x=y*.

**Proof.**

One has *w*′′(*r*)=(*γ*−1)*r*^*γ*−2^−(*α*−1)*r*^*α*−2^>0, for all *r*>0. Thus, *w* is strictly convex. Then, one has, by the strict convexity of *w*,

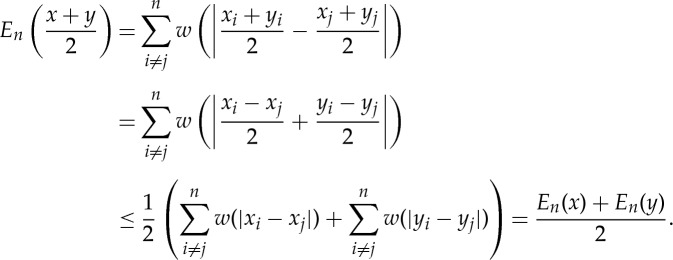

The equality above is strict unless *x*_*i*_−*x*_*j*_=*y*_*i*_−*y*_*j*_ for all *i*,*j*, that is *x*_*i*+1_−*x*_*i*_=*y*_*i*+1_−*y*_*i*_. Therefore, *x*=*y*. ▪

As a consequence, we can now state the following result regarding the uniqueness of global discrete minimizers.

**Theorem 3.3**

*Suppose that α≤1, γ≥1 and α<γ. Up to translations, the minimizer x of E*_*n*_ *is unique and symmetric with respect to its centre of mass.*

**Proof.**

Let *x*, *y* be two minimizers of *E*_*n*_ satisfying (3.3). If *x*≠*y*, by lemma 3.2, one has

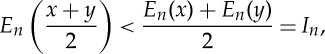

and a contradiction. This shows the uniqueness of a minimizer satisfying (3.3a). Denote now by *s* the symmetry defined by *s*(*ξ*)=−*ξ*, 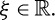 If *x* is a minimizer of *E*_*n*_(*x*) satisfying (3.3a) then *y* defined by

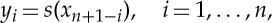

is also a minimizer satisfying (3.3b). Thus, by uniqueness,

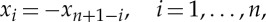

and this completes the proof of the theorem. ▪

**Remark 3.4 (uniqueness and displacement convexity in one dimension)**

Lemma 3.2 and theorem 3.3 are just discrete versions of uniqueness results for the continuum interaction functional (1.1). In the seminal work of McCann [45] that introduces the notion of displacement convexity, he already dealt with the uniqueness (up to translation) of the interaction energy functional (1.1) using the theory of optimal transportation: if *W* is strictly convex in 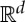, then the global minimizer is unique among probability measures by fixing the centre of mass, as the energy *E*[*μ*] is (strictly) displacement convex. However, the displacement convexity of a functional is less strict in one dimension than in higher dimensions. As proved in [46], to check the displacement convexity of the energy *E*[*μ*] in one dimension, it is enough to check the convexity of the function *w*(*r*) for *r*>0. Therefore, if *w*(*r*) is strictly convex in 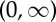, then the energy functional (1.1) is strictly displacement convex for probability measures with zero centre of mass. As a consequence, the global minimizer of (1.1) in the set of probability measures is unique up to translations. Lemma 3.2 shows that this condition is equivalent to *α*≤1, *γ*≥1 and *α*<*γ*, for power-law potentials. Finally, the convexity of *E*_*n*_ in lemma 3.2 is just the displacement convexity of the energy functional (1.1) restricted to discrete measures. We included the proofs of the convexity and uniqueness because they are quite straightforward in this case, without appealing to more involved concepts in optimal transportation.

**Remark 3.5 (explicit convergence to uniform density)**

As a final example, we consider the case where *γ*=2, *α*=1, which corresponds to quadratic attraction and Newtonian repulsion in one dimension (see [6]). When *x* is a minimizer of *E*_*n*_(*x*), we have by (2.7) that

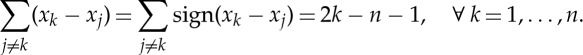

Replacing the index *k* by *k*+1, the equation becomes

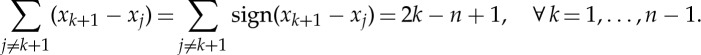

Subtracting the two equations above, we obtain

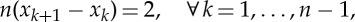

that is *x*_*k*+1_−*x*_*k*_=2/*n*, for all *k*=1,…,*n*−1.

This shows that, in the case *γ*=2 and *α*=1, the points *x*_*i*_ are uniformly distributed; as *n* goes to infinity, the corresponding discrete measure 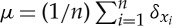 converges to the uniform probability measure on the interval [−1,1]. This uniform density is known to be the global minimizer of the energy *E*[*μ*] in the continuum setting; see [6,27].
